# 90. Interim Assessment of Safety and Immunogenicity From a Proof-of-Concept Phase 2 Trial of an mRNA-Based Cytomegalovirus Vaccine in Patients Who Have Undergone Allogeneic Hematopoietic Cell Transplantation

**DOI:** 10.1093/ofid/ofaf695.036

**Published:** 2026-01-11

**Authors:** Nicolas C Issa, Jessica S Little, Jennifer Husson, Chunla He, Benjamin Lorenz, Lindsey R Baden, Lori Panther

**Affiliations:** Brigham & Women's Hospital, Boston, Massachusetts; Brigham and Women's Hospital, Boston, MA; Moderna, Inc., Cambridge, Massachusetts; Moderna, Inc., Cambridge, Massachusetts; Moderna, Inc., Cambridge, Massachusetts; Brigham and Women's Hospital, Boston, MA; Moderna, Inc., Cambridge, Massachusetts

## Abstract

**Background:**

Cytomegalovirus (CMV) establishes lifelong latency and is a risk factor for increased mortality in immunosuppressed individuals. mRNA-1647, an investigational mRNA-based vaccine targeting CMV gB and pentamer antigens, demonstrated acceptable safety and generated antigen-specific humoral and cell-mediated immunogenicity in healthy adults. We present interim analyses of mRNA-1647 safety and immunogenicity from an observer-blind phase 2 trial (NCT05683457) in CMV-seropositive adults ≥18 years with prior allogeneic hematopoietic cell transplantation (HCT).

**Figure:**
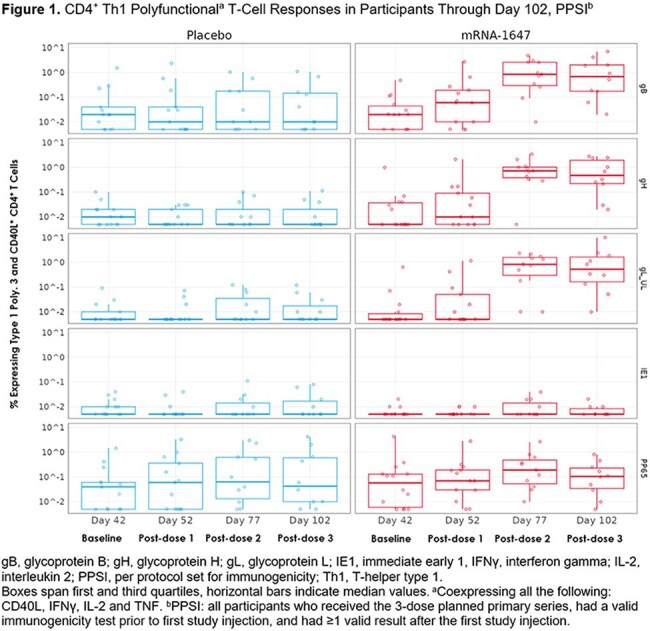

**Figure:**
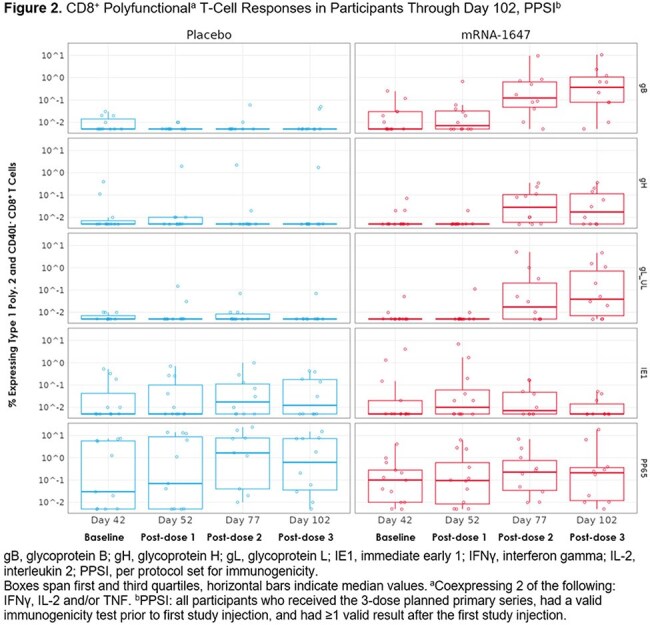

**Methods:**

Participants were randomized 1:1 to receive a 3-dose primary series of mRNA-1647 150 μg or placebo on Days 42, 67, 92 post-HCT, prior to the critical risk period for CMV reactivation (Day 100 post-HCT and/or CMV prophylaxis cessation). Safety was a primary endpoint. Humoral immunity (secondary endpoint) was measured by cell-based neutralizing antibody (nAb) assays at baseline and 25 days after each dose. CMV-specific T cell responses (gB- and pentamer-specific) were assessed as exploratory endpoints by intracellular cytokine staining and polyfunctionality analyses at baseline and 10 days after each dose. Site personnel remain blinded to safety analyses.

**Results:**

At data cutoff (7 May 2024), 44 participants were randomized to receive mRNA-1647 or placebo (n=22/group; median age 65 years, 51.2% male, 72.1% White). There were no substantial differences between groups in occurrences of acute or chronic graft-vs-host disease, disease relapse, or serious adverse events. In the mRNA-1647 group vs placebo, nAb GMTs against epithelial cell infection increased ∼2.2-fold after dose 2 and ∼3.2-fold after dose 3. nAb titers against fibroblast infection remained similar between groups. mRNA-1647 induced robust CD4^+^ and CD8^+^ T cell responses against CMV-specific glycoproteins (gB, gH, gL) after dose 2, with T cells exhibiting polyfunctionality of substantial magnitude (Figures 1, 2).

**Conclusion:**

In high risk seropositive HCT recipients, mRNA-1647 increased nAbs against epithelial cell infection and demonstrated antigen-specific, polyfunctional CD4^+^ and CD8^+^ T cell responses. There were no safety concerns on blinded safety assessment. These data support the continued assessment of mRNA-1647 in this population.

**Disclosures:**

Jessica S. Little, MD, Merck and Company, Inc.: Grant/Research Support|Moderna, Inc.: Grant/Research Support

